# Telehealth Research Strategies in a Post COVID-19 Era: A Roadmap for the Coming Decade

**DOI:** 10.1089/tmj.2024.0520

**Published:** 2024-12-06

**Authors:** Lorraine R. Buis, Christopher McGovern, Charles R. Doarn

**Affiliations:** ^1^ Department of Family Medicine, University of Michigan, Ann Arbor, Michigan, USA.; ^2^ Connected Nation, Inc., Bowling Green, Kentucky, USA.; ^3^ Department of Environmental and Public Health Sciences, College of Medicine, University of Cincinnati, Cincinnati, Ohio, USA.

**Keywords:** telemedicine, telehealth, research, pandemic, COVID, roadmap

## Abstract

An Agency for Healthcare Research and Quality-funded Think Tank, held at Michigan State University, brought together a wide range of subject matter experts in telemedicine, telehealth, digital health, digital access, and health care. The authors of this article represented a group focused on the research needs, constructs, and strategies in the post-COVID era. While telemedicine and telehealth grew exponentially during the pandemic, the challenges that have been with us for decades, while ameliorated to some extent, remain. To showcase the State of Michigan as a model, the authors reviewed challenges and opportunities related to telehealth and telemedicine usage and categorized them into seven areas of highest priority. Based on a review of the literature and consultation in the fields of telehealth, telemedicine, and digital access, we identified seven key categories where research would be most effective going forward. These categories include research into the impact of telehealth on clinical services, telehealth’s use in administrative activities, education and training for health care providers and patients, telehealth policy implications at state and federal levels, the impact of future technology and innovation on telehealth services, patient characteristics and their experiences, and the ethical aspects of telehealth in the future. We have formulated this overview of our findings to act as a roadmap for future telehealth research. We recommend that ongoing studies should explore each of the seven categories identified above. The search for solutions to overcome the challenges within these topics must not be constrained to research that has been conducted recently; many of these challenges have faced telehealth researchers for decades, and numerous solutions have been proposed over the years. Some of these proposals should be explored once again in light of technological and societal advances. The findings from these studies should be shared in ways and through venues that can make them accessible outside the spheres of academic research, but also by practitioners, policymakers, and patients. Third, research into telehealth in all its incarnations must be prioritized, and leadership is needed to ensure these areas of study are continued to be spotlighted after the noise of the COVID-19 pandemic grows quiet.

## Introduction

Despite decades of research, demonstration projects, and investment in the field of telemedicine and telehealth, widespread adoption of synchronous and asynchronous telehealth within routine clinical care still needs to be improved. The COVID-19 pandemic, however, forced a rapid expansion of telehealth in the United States (U.S.) and across the globe.^1,2^ During the Public Health Emergency (PHE), telehealth was thrust into the spotlight for its ability to connect patients with their care teams in meaningful ways when access to health care services had been reduced.^3^

Swift and broad federal emergency policy changes allowed for increased telehealth utilization. Policymakers and insurers relaxed reimbursement and regulatory policies to expand telehealth access to more patients. They granted payment parity, allowing providers to bill telehealth visits at the same rate as in-person visits.^4^ Moreover, the federal government invested funding to support rapid telehealth expansion for the benefit of all.

Americans see the potential benefits of telehealth. Since the pandemic began in 2020, we’ve had ample opportunity to see where we lack data about telehealth access and usage. Although telehealth utilization has waned somewhat since its height early in the pandemic, it remains an important tool that allows health care to be accessed in ways that were previously impossible for many Americans. As Rokosh et al. posited, we are truly living in a time of clinical transformation.^5^

## Methods

In August 2024, an Agency for Healthcare Research and Quality (AHRQ)-funded Think Tank brought subject matter experts and practitioners with expertise in policy, clinical care, clinical education, technology, patient equity, and research, to Michigan State University to discuss the history and future of telehealth in Michigan and beyond. This 3-day event generated high quality discussion and collaboration from the roughly 30 experts invited to take part. Organizers gathered participants into six groups, each with a specific focus.

The organizers charged our group with reviewing research needs. Specifically, we were tasked with identifying and making recommendations on areas of future research that will shape the telehealth conversation in the future. These recommendations would then be the seed for a research agenda to identify ways to improve telehealth opportunities in the post-COVID era.

Strengthened by the work of previous similar gatherings, this paper is the result of a subgroup in the Think Tank focused on exploring the current state of telehealth research.^6–10^ Our goal is to set forth a research agenda that supports the expansion of future telehealth research. With this goal in mind, our subgroup identified seven research themes and examples of questions that can guide future telehealth research. These themes provide a foundation for the short- and midterm research agenda needed for the continued advancement of telehealth over the next decade and beyond.

## Results

Through discussions within our subgroup and other Think Tank participants, along with an analysis of existing literature in the field of telehealth research, our group delineated seven thematic areas with opportunities for focus in future telehealth research: (1) clinical, (2) administrative, (3) education and training, (4) policy formulation, (5) technology and innovation; (6) patient characteristics and experience opportunities, and (7) ethics.

While we identify unique issues for each of these seven research domains, there are some issues that cut across all telehealth research. For example, for all seven fields of research, leadership is essential to guide the way that data are collected and disseminated. More importantly, for every area of research that must be plumbed, there must be a devotion to exploring the decades of studies that have come before.^11^ Though research on telehealth has grown significantly since the COVID-19 pandemic, the study of telehealth does must continue as we move forward. Many of the same issues that are being explored today have been explored and written about in the last century.^12^ Solutions that were suggested then may once again be relevant and are ignored at our own peril.

## Clinical Opportunities

Telehealth can be applied in every clinical discipline, and technological capabilities often dramatically change clinical care.^13^ As telehealth becomes accepted by more practitioners and patients, research is needed to understand how best to optimize its use within a clinical setting.^14^ Finding the right use cases, technologies, work flows, and processes will maximize returns and provide better patient clinical care. Clinical workflows that work for face-to-face encounters may not be the most efficient for virtual encounters; other possibilities, such as the creation of new job roles and classes, may work best to optimize telehealth.

Concerns must be addressed about whether the health care industry will phase out face-to-face care in favor of telehealth approaches. Despite its ability to streamline some services, telehealth remains only one tool in the full suite of clinical care options. Finding the right way to use this tool can streamline costs and improve patient care.

## Administrative Opportunities

Often telehealth has been stymied by administrative challenges. These challenges include a lack of understanding, lack of data, trustworthiness, cost burdens, and very often leadership.^15^ Incorporating telehealth into administrative tasks can provide financial savings, improved workflow, and more efficient services for patients. Research can help plan for the changes that telehealth technologies will undoubtedly have in store for administrators.

A comprehensive needs assessment is needed—what are the goals for incorporating telehealth technology into the administrative milieu? It could be to streamline businesses, assess (or redefine) returns on investment, reduce costs, or reduce waste. This could include current definitions of success, but the ability for telehealth to transform administrative duties means success metrics may need to be reevaluated.

Research in this field should not neglect the work that has come before. Any effort to identify future opportunities should be cognizant of prior research, along with collaborations with those who may address problems from a different angle or attempt to reach these same goals for other purposes. Intellectual discourse and disagreement help move us forward.

## Education/Training Opportunities

Attention must be devoted to understanding how best to teach health care providers how and when to utilize telehealth modalities. Learning how technological biases or other discriminatory processes may affect diagnostic results is crucial to assure that every patient gets the best possible care. This knowledge must then be built into curricula for new health care providers and part of continuing medical education for those already on the job. Research can help identify the education necessary for technicians, support personnel, IT professionals, etc., and best practices for delivering that education.

Patients must also be educated. Teaching patients how and when telehealth can and should be used is an important part of streamlining care. Patients who schedule virtual visits with a health care provider should be educated on what types of encounters make most sense for using this modality. This education must always be inclusive and culturally relevant, keeping cultural context and historic experiences with the health care community in mind.

## Policy Opportunities

We live in an exciting and uncertain time for telehealth. Despite emergency regulatory and reimbursement policy changes, these changes have not yet been made permanent. Although we do not anticipate that telehealth will go away, what happens at the policy level has the potential to have an enormous impact on telehealth utilization in the future.

Researchers can measure the impact of policy changes on telehealth acceptance and usage. Identifying which policies have benefitted patients, and which ones have failed to result in significant positive change, is important as policymakers plan the future policies that will expand (or limit) telehealth access.

Currently, too much information about telehealth is being disseminated by policy groups, lobbyists, and other entities with their own agendas. Unbiased, vetted, quality research, presented in ways that can be understood by those who make health care policy, is a crucial component for making informed policy decisions.

## Technology and Innovation Opportunities

Within the field of telehealth, it is easy to focus solely on virtual visits; however, a wide variety of future technologies are ripe for research. Remote patient monitoring is gaining more momentum as the remote collection of patient-generated data becomes easier to obtain and is more often integrated into electronic health records. Telecommunication modalities are constantly improving, as is high-speed internet access in rural areas. Reimbursement and regulatory policies are starting to support this more broadly, which will be critical as hospital-at-home programs expand, while artificial intelligence and robotics are at the cutting edge of telehealth opportunities.

Work is also needed to optimize the user experience for patients and providers, particularly culturally sensitive solutions for a variety of patients with differing levels of digital literacy and health literacy. Better user experience will likely lead to better patient engagement, potentially improving health outcomes.

## Patient Characteristics/Experience Opportunities

The COVID-19 pandemic pulled back the curtain on many patient factors that hadn’t been addressed widely. Issues like digital literacy, access, medical mistrust, and systemic racism, have yet to be addressed.

Identifying the best steps to improve patient acceptance and awareness of telehealth services is crucial for increased adoption of these modalities. Using both qualitative and quantitative methodologies, we must identify the digital skills that patients need, as well as how best to familiarize patients with telehealth options and their use. While traditional patient education modalities, such as written materials and videos may represent low-hanging fruit for educating patients, newer innovative approaches are currently also being explored such as digital navigators to provide patient assistance. Learning how to effectively help patients find and utilize telehealth tools is crucial to improving their usage; are patients aware of (or do they care about) differences between services provided by their established health care provider, vs. a direct-to-consumer online dispensary? Designing telehealth tools in a culturally sensitive manner to address the specific needs of different patient communities and overcome their unique challenges will be needed.

## Telehealth Ethics

Finally, there exist multiple ethical concerns that research should address.^16^ Data stewardship and risk mitigation are important when patient data is involved. Determining how best to collect high-quality data while ensuring that only the minimum information needed is gathered is crucial. Moreover, what is the responsibility of health care providers to monitor and act upon this data in a timely fashion? Ethical uses of data, how that data can be used, and data retention policies are all important questions to be answered, and those answers will vary between projects and patient populations.

A related question is whether data is sufficient to show conclusive results, or are researchers “jumping the gun” to remain timely? Reproducibility in research is important, but this can be difficult to achieve with human subject populations. When does the need for reproducibility hinder the obligation to share important information with the research community?

Answering these questions is crucial in today’s climate of distrust toward health care institutions. A negative experience could adversely impact a patient’s view of telehealth. As such, identifying and addressing the ethical challenges related to telehealth must be prioritized.

## Conclusions

One thing that was made clear from this Think Tank is that the questions telehealth researchers and policymakers have been grappling with for the past several decades are still the questions we are trying to solve today. Issues of reimbursement, licensure, security and confidentiality of data, bandwidth, and opinions, while ameliorated to some degree, remain barriers. The pandemic accelerated the application of telehealth and no one expects it to be removed as a significant adjunct in health care delivery. Much has been learned and much has yet to be understood. As technology continues it unabated growth, so too is our insatiable desire and appreciation for better health care. These seven areas will undoubtedly help us all continue championing change and moving this field further.
